# Extending the Framework for Developing Intelligent Virtual Environments (FIVE) with Artifacts for Modeling Internet of Things Devices and a New Decentralized Federated Learning Based on Consensus for Dynamic Networks

**DOI:** 10.3390/s24041342

**Published:** 2024-02-19

**Authors:** Miguel Rebollo, Jaime Andrés Rincon, Luís Hernández, Francisco Enguix, Carlos Carrascosa

**Affiliations:** 1 Valencian Research Institute for Artificial Intelligence, Universitat Politècnica de València, 46022 Valencia, Spain; lhernand@dsic.upv.es (L.H.); fraenan@upv.es (F.E.); carrasco@dsic.upv.es (C.C.); 2 Departamento de Digitalización, Escuela Politécnica Superior, Universidad de Burgos, 09200 Miranda de Ebro, Spain; jarincon@ubu.es

**Keywords:** complex networks, distributed AI, multi-agent systems (MASs), neural networks

## Abstract

One of the main lines of research in distributed learning in recent years is the one related to Federated Learning (FL). In this work, a decentralized Federated Learning algorithm based on consensus (CoL) is applied to Wireless Ad-hoc Networks (WANETs), where the agents communicate with other agents to share their learning model as they are available to the wireless connection range. When deploying a set of agents, it is essential to study whether all the WANET agents will be reachable before the deployment. The paper proposes to explore it by generating a simulation close to the real world using a framework (FIVE) that allows the easy development and modification of simulations based on Unity and SPADE agents. A fruit orchard with autonomous tractors is presented as a case study. The paper also presents how and why the concept of artifact has been included in the above-mentioned framework as a way to highlight the importance of some devices used in the environment that have to be located in specific places to ensure the full connection of the system. This inclusion is the first step to allow Digital Twins to be modeled with this framework, now allowing a Digital Shadow of those devices.

## 1. Introduction

This paper is an invited extension of the paper titled “GTG-CoL: A new Decentralized Federated Learning based on Consensus for Dynamic Networks” [1].

Wireless Ad-hoc Networks (WANETs) are composed of a set of mobile units (agents) that are equipped with wireless antennas, allowing them to communicate with each other in a decentralized manner [2]. These devices may have limited communication range and battery life, and as the agents move dynamically in the environment, they create a switching topology of the communication network graph. For this reason, this scenario can be defined as a Geographical Threshold Graph (GTG)  [3,4], according to the maximal scope given by the agents’ antennas. Therefore WANETs can be applied to collaborative sensors in (autonomous or human-driven) tractors that operate in rural areas or remote locations with limited connectivity.

When situated in rural areas far from connectivity availability and with the problems of being executed during a long period without a person controlling them or assuring their battery supply, these fields define a new interesting problem where AI techniques can be applied to adapt to those problems. One of the challenges of WANETs lies in enabling distributed learning among the agents without relying on a central server or sharing their private data. Federated Learning (FL) is a machine learning technique that allows multiple agents to collaboratively train a model while keeping their data locally [5]. However, most FL algorithms assume a centralized coordinator that aggregates the local models from the agents, which is not desirable in WANETs. Therefore, decentralized FL algorithms that use consensus mechanisms to achieve agreement on the model parameters among the agents are more suitable for WANETs.

Research on cooperating distributed systems, which is particularly important in the distributed learning area, has increased in recent years. One of the approaches of decentralized FL based on consensus in multi-agent systems is the CoL [6] algorithm. CoL enables the agents to share and aggregate their learning models with their direct neighbors within their antenna range, using a gossiping protocol. CoL can be applied to train neural networks for various tasks, such as fruit disease prediction, without compromising data privacy or network efficiency.

The paper aims to study the application of consensus in multi-agent systems (using the CoL algorithm) to this Geographical Threshold Graph to allow us to model the scenario described above. Apart from the theoretical analysis, the paper presents the application of these graphs to FIVE, a Framework for developing Intelligent Virtual Environments inhabited by SPADE agents [7]. This framework allows for quickly defining or modifying a simulation, where a deployment of a set of mobile agents can be tested in a simulation of a crop field before deploying them in the actual environment.

In addition, the paper presents the inclusion of artifacts in the FIVE framework. Artifacts can provide services and functions to the agents, such as data storage or communication. This allows us to model scenarios such as the one described above, where IoT devices and other static elements of the environment are located in specific places to study the full connection of the system and to enable the development of a Digital Shadow (DS) of the environment. A DS is a virtual representation of the environment that synchronizes data from the real world in real-time.

The following section presents a state-of-the-art of the leading research lines in the work. Next, GTG-CoL as the application of CoL algorithm for the Geographical Threshold Graph is presented. After that, the Orchard Digital Model designed for validating the WANET designs is presented along with a section detailing a simulation of fruit orchard smart areas using the given framework. Finally, a section describes how and why artifacts have been included in the FIVE framework. The paper finishes with some conclusions and comments about future work.

## 2. State of the Art

This section introduces the different subjects or areas related to the work presented in the paper: artifacts, as the concept to model the static parts of an environment; Intelligent Virtual Environments (IVEs) [8] as virtual environments simulating a physical (or real) world inhabited by intelligent agents; Digital Twins (DT); Federated Learning and consensus in multi-agent systems.

### 2.1. Artifacts

According to the A&A meta-model,  [9] for MAS, when defining a MAS and its environment, all the different parts of the system can be defined in terms of agents and artifacts. Agents are the autonomous parts of the system, committed to achieving the goals and tasks defining the whole MAS behavior reactively and proactively. On the other hand, artifacts are the passive, reactive parts allowing to model the environment with the services and functions needed and used by the agents.

One of the key challenges in modeling and engineering agent societies and MAS environments is the need to represent the complex interactions between agents and their environment [10]. Artifacts are objects that can be used to represent knowledge, information, or data in MAS. They provide a way to represent these interactions in a more structured and formal way, which can help improve MAS’s efficiency and effectiveness [11].

The use of artifacts in MAS has been applied to many research fields, including robotics, artificial intelligence, and distributed systems. For example, artifacts have been used to represent the environment in which robots operate, as well as the interactions between robots and their environment. Artifacts have also been used to describe the interactions between agents in distributed systems, such as the IoT [12,13].

Artifact frameworks provide a standardized way of representing resources and reduce the amount of code that needs to be written. By using artifact frameworks, researchers can focus on the high-level design of their MAS rather than the low-level implementation details of individual agents. This can lead to more efficient and effective development processes.

CArtAgO is one such artifact framework that provides a set of artifacts to facilitate the development of agents. It is meant to be integrated and exploited with external agent frameworks or platforms, particularly those that adopt Java as the underlying implementation language [14,15]. The JaCaMo platform combines Jason for programming autonomous agents, CArtAgO for programming shared environments, and Moise for programming agent organizations [10]. The JaCaMo framework is also used in developing mobile apps as personal assistant agents [16]. RV4JaCa is a runtime verification approach for MAS using the JaCaMo framework that improves the security level controlling events during the execution of the system without needing a specific implementation in the behavior of each agent to recognize the events [17]. Using the artifact frameworks, developers can create more flexible and dynamic MAS to adapt to the environment-changing conditions at runtime.

### 2.2. Intelligent Virtual Environments

In the evolving landscape of technology, Intelligent Virtual Environments (IVEs) offer a dynamic convergence of advanced environment simulations and artificial intelligence (AI). At the heart of IVEs lies the integration of intelligent agents, entities endowed with cognitive capabilities that navigate and interact within a virtual realm. The integration of virtual environments with intelligent agents capable of interacting within them presents opportunities to simulate realistic scenarios and facilitates the execution of decision-making simulations [18].

These agents, driven by sophisticated algorithms and AI, contribute to creating a virtual space that replicates the physical world and responds dynamically to user input and environmental changes. The inclusion of intelligent agents into the virtual environment introduces a level of complexity beyond static simulations, offering a practical understanding of how systems, entities, and users interact within the digital domain. This technology finds applications across diverse sectors, from agriculture [19] to industries such as robotics, IoT, and urban planning [18].

Flexible IVE designer (FIVE) is a framework that uses Unity3D and SPADE agents [20]. It allows the rapid and easy creation of custom IVEs by importing 3D models of the elements of the IVE. Then, we can create the IVEs by modifying text files. Currently, it counts with a user-helper tool that enables the automatic generation of a 3D virtual environment by selecting a real area on an interactive satellite map. The JaCalIVE framework is for developing and simulating IVEs based on the MAM5 meta-model and the A&A paradigm [21]. It integrates agents, artifacts, and physical simulation to create realistic and complex IVEs. MAMbO5 is an ontology that merges the concepts of MAM5 and OOVASIS and adds new concepts to enhance the expressiveness and flexibility of the model [22]. MAMbO5 can be used as an input of JaCalIVE, and it allows for representing IVEs as situated and non-situated organizations with roles, strategies, processes, and other organizational aspects.

### 2.3. Digital Twins

The concept of Digital Twins (DTs) describes a real-time synchronized virtual representation of a product, process, or environment [23]; this includes both a physical entity and a virtual counterpart and the connections between them [24]. Depending on the level of data integration, Kritzinger [25] presents three levels of integration:

- Digital Model: there is no automatic data exchange between the physical and virtual environments.
- Digital Shadow: information flows from the physical to the virtual world.
- Digital Twin: where information flows in both directions and changes in one world affect the other. A digital twin would therefore be a computer system that accurately reflects a physical object or process and is capable of reacting in real time to the same inputs that its real twin receives, undergoing the same changes and producing the same responses as its real twin. In a way, a digital twin would be interchangeable with its real model in studying its behavior and in decision making.

A review of the current state of Digital Twin research and a roadmap for its evolution can be found in [26]. Research trends in Digital Twins are varied. On the one hand, we can see the different technologies being applied in this field to improve the capabilities of DT, such as AI, distributed computing, 5G, virtual reality, or IoT [27]. On the other hand, we can see the ever-widening field of application areas such as energy control systems for fault detection and energy saving and processes related to manufacturing such as transportation and cities [28]. The area that concerns us, agriculture, is also undergoing a necessary evolution to meet the challenges of increased population or climate change. The use of technology is increasing, and the use of digital twins for greater efficiency is of growing interest [29]. Current research on the use of digital twins in agriculture covers different types of applications that Purcell in [30] classifies as:

- Crops: monitoring, resource optimization, and cultivation support.
- Urban: controlled environment and aquaponic farming.
- Livestock farming: monitoring, management, and optimization.
- Product design: smart services and machinery management.
- Supply and value chains: This includes environmental condition management or the use of DT to evaluate and improve the performance of value chains.
- Policy, environment, and infrastructure: DTs to facilitate policy decisions related to agriculture based on data collected in real time.

### 2.4. Federated Learning

Federated (machine) learning (FL) is an ML technique that enables multiple devices to collaboratively train a model without sharing their data with a central server [5]. Integrating FL with MAS can enable agents to share knowledge collaboratively and cooperatively.

Lately, there has been a lot of work in applying FL and new lines of research related to it. More specifically, FL’s application areas include but are not limited to the following: autonomous vehicles, recommender systems, fintech, IoT, mobile services [31], and medical applications [32]. Regarding the lines of research in FL, they are wide and diverse; the latest developments include the following:

- Application of FL to IOT devices [33].
- Improving the aggregation of FL models [34].
- Evolving the centralized approach of FL to a decentralized one [35,36,37].
- Mixing FL with blockchain [38] and in [39].
- Techniques for clustering FL participants [1,40,41].
- Application of model optimization technologies to FL [42].
- Improving communication, such as ensuring data privacy or reducing network overhead [43,44].
- Integration of Deep Reinforcement Learning (DRL) with FL enables agents to learn from each other’s experiences and improve their performance collaboratively. This integration can also improve the efficiency and quality of Mobile-Edge Computing (MEC) [45,46].

### 2.5. Consensus in Multi-Agent Systems

Consensus, within the context of MAS, refers to the attainment of a unified value or agreement on overall intelligent agents through communication and collaboration among individual agents. In distributed systems, consensus algorithms, such as the one introduced by Olfati-Saber and Murray [47], play an essential role in achieving agreement on a singular data value among distributed processes or systems. These algorithms are commonly employed in scenarios where a group of agents, each possessing its private estimate of a shared parameter, attempt to reach a consensus on the parameter’s value through local interactions.

This concept has garnered substantial attention within scientific communities, leading to the development of numerous algorithms aimed at ensuring that agents converge toward a consensus value [48,49].

Following the aforementioned definition presented by Olfati-Saber and Murray, a consensus process in a MAS is a problem where the agents reach an agreement about the value of a variable of interest without any intermediate or leader that rules the process. It is an iterative procedure. The agent $a_{i}$ calculates the new value $x_{i}\left(t+1\right)$ in each iteration, according to Equation (1).

(1) $$x_{i}\left(t+1\right)=x_{i}\left(t\right)+\sum_{j\in N_{i}}\left[x_{j}\left(t\right)-x_{i}\left(t\right)\right]$$

where $N_{i}$ denotes the neighbors of agent $a_{i}$, and ε is the learning step: a factor bounded by the maximum degree of the network. The consensus converges to the average of the initial values $\langle x_{i}\left(0\right)\rangle$ whenever $\varepsilon \leq \frac{1}{\max d_{i}}$. Olfati-Saber and Murray have demonstrated the convergence of the process, even with time delays or switching topologies, allowing agents to change their neighborhood dynamically.

**Figure 1:**
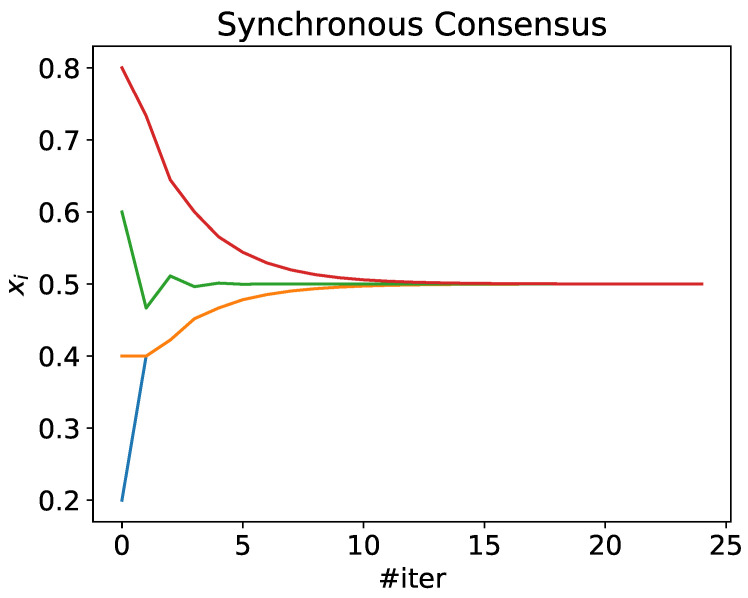
Consensus evolution in a network with four agents. Each line represents the evolution of $x_{i}\left(t\right)$ for agent *i*. Initially, x(0)={0.2,0.4,0.6,0.8}, so 〈x(0)〉=0.5.

As an example, Figure 1 shows a simple case with a network formed by four agents. The initial values are x(0)={0.2,0.4,0.6,0.8}. Each individual agent updates its value using Equation (1). After 25 iterations, all the values have converged to 0.5, which is the average of the initial values $x_{i}\left(0\right)$ of each agent.

While both FL and consensus algorithms involve distributed computation and communication among multiple agents or nodes, they serve distinct purposes and operate differently. FL is explicitly crafted for training machine learning models in a decentralized and privacy-preserving manner. Conversely, consensus algorithms are versatile tools designed for achieving agreement in distributed systems.

It is noteworthy that some FL algorithms incorporate elements of consensus algorithms to ensure the convergence of local models toward a global model [20]. However, this integration is not inherent to the FL framework and is not universally applied across all FL algorithms. The synergy between consensus and FL highlights the adaptability and versatility of these approaches, offering researchers and practitioners a spectrum of tools to address diverse challenges in distributed intelligent systems [50].

## 3. GTG-CoL

### 3.1. Problem Definition

We apply CoL in WANETs, where a vast area has limited connectivity. At design time, it is essential to ensure that the system will eventually form a strongly connected component for distributed systems to propagate the information among the participants using gossiping mechanisms. We want to apply FL to train neural networks without central servers, sharing the weights and biases with the direct neighbors. First, we present our decentralized FL algorithm, *CoL*, and how it can be applied to WANETs. Next, we develop how it can be assured at design time that a strongly connected component emerges from the MAS.

### 3.2. Co-Learning Algorithm

**Figure 2:**
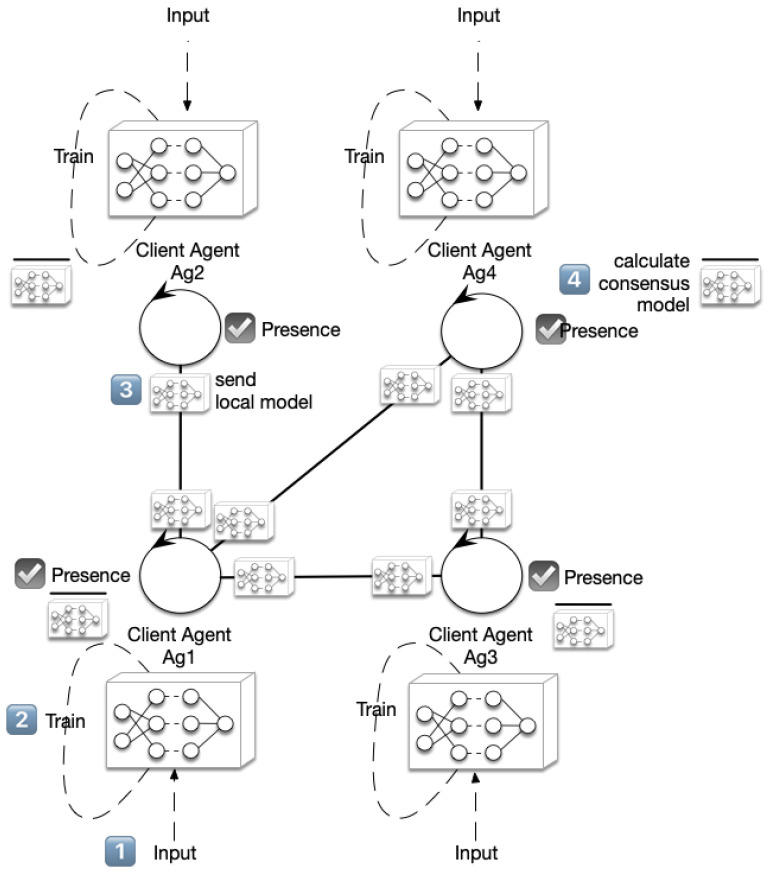
MAS for FL through a consensus process.

The Co-Learning (*CoL*) algorithm [6] is a decentralized FL algorithm that uses a consensus in a multi-agent systems approach to share the weights of the neural network model each agent is learning, taking into account that all agents share the same neural network structure (see Figure 2). This allows the agents to share the model being learned to aggregate with the others’ models so that a new aggregated model can be used instead of their local one.

In this case, the sharing is made only with the local neighbors of each agent, applying the consensus equations to obtain the aggregated model. Those weights can be propagated based on consensus properties, and all the agents will converge to their average. The complete model’s weights and biases can be considered as independent variables from the consensus point of view. Therefore, we can assume that the process comprises *n* parallel consensus or one consensus over a vectorized variable with *n* components, with *n* being the total number of parameters of the neural network.

Algorithm 1 details the steps an agent ai following the Co-Learning process. Áll agents share the same structure for the deep learning network. It is formed by a set of *k* layers with different purposes (such as embedding, convolution, or LDST). Without losing generality, we can assume that the model is a set of weights and bias matrices $W=(W_{1},\ldots ,W_{k})$. The particular values for *W* will depend on the dataset each agent has available and the training process executed. These steps can also be observed in Figure 2: first of all, it will train the neural network during *e* epochs (steps 1 and 2, input and training, in Figure 2). This training results in the set of the *k* matrices stored in *W*. For each one of them, the agent iterates *c* times over the consensus phase, leading to *k* new matrices that will be used in the training process again (step 4, calculate consensus model, in Figure 2). This consensus is calculated with the models received from $a_{i}$ neighbors, to which $a_{i}$ has previously sent its local model (step 3, send local model, in Figure 2).
**Algorithm 1** $CoL_{a_{i}}\left(e,k,c\right)$ —Co-Learning Algorithm for agent $a_{i}$
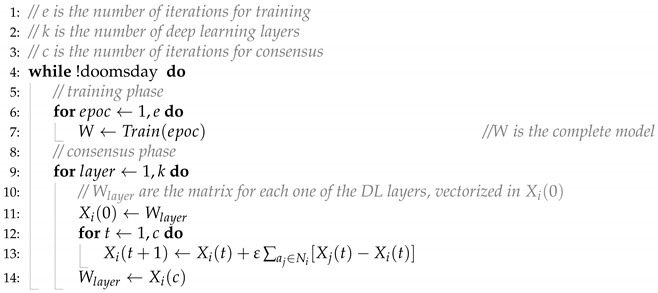


The process is executed in parallel as many times as the parameters of the artificial neural network have. Applying CoL algorithm to agents in WANET can be made by adapting it to take into account the switching topology of the network; that is, the set of neighbors of each agent $N_{i}$ may change dynamically (as the agent could only communicate with the ones inside the range of their antenna. So, in the model, $N_{i}\left(t\right)$ has to be considered. Even in this case, the convergence of the model is ensured whenever the agents’ network eventually forms a unique connected component [47].

### 3.3. Network Characterization for WANETs

The network topology does not affect the result of the consensus process but does the convergence speed. A strong assumption is that all the participants must eventually form one connected component. Olfati-Saber and Murray [47] have demonstrated the algorithm’s convergence under switching topology.

Carrascosa et al. [6] have studied different network topologies. This work concludes that random geometric graphs (RGGs) are the most appropriate topology for a set of agents to coordinate. Existing networks following the same structure are known as Geographical Threshold Graphs (GTGs), so this is the network topology preferred for the agent connection. For the theoretical study, and without losing generality, we project the geographical space occupied by the orchard into a square area in a ${\left[0,1\right)}^{2}$ space. The agents will follow a set of rectilinear paths at regular distances. As a test bed, we define three different configurations:

**Test 1:**  one orchard with all robots running over parallel end-to-end lines.
**Test 2:**  two orchards next to each other, with agents moving perpendicularly.
**Test 3:**  Test 2 + one extra agent that moves following a random walk.
**Test 4:**  one orchard with all agents moving following a cyclic random walk.
**Test 5:**  Test 4 + a static network of beacons.

**Figure 3:**
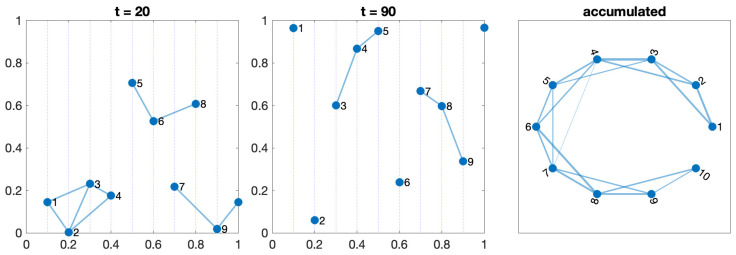
Network evolution with ten robots moving along lines in an orchard at different speeds.

**Figure 4:**
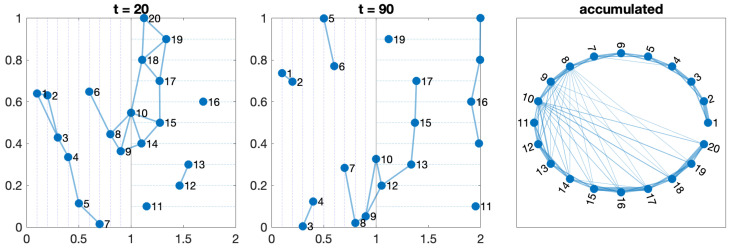
Network evolution with ten robots in each one of the two orchards moving along perpendicular lines.

**Figure 5:**
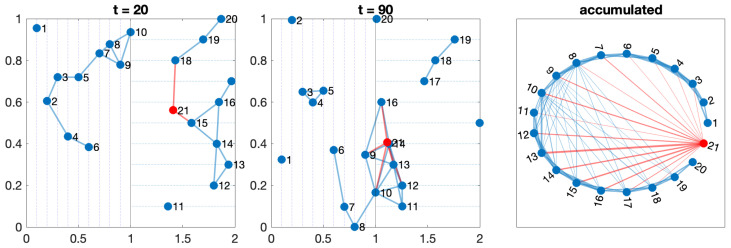
Network evolution with 20 robots in two orchards moving along perpendicular lines (in blue) and one drone moving freely following a random walk (in red).

**Figure 6:**
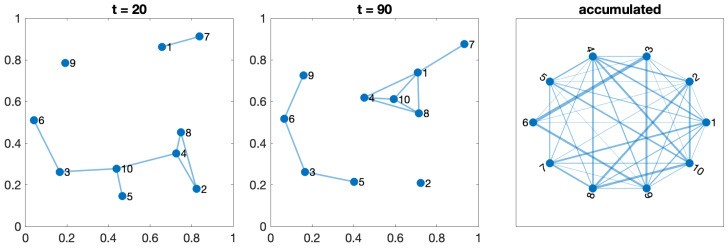
Network evolution with ten drones moving freely following cyclic random walks with different periods.

**Figure 7:**
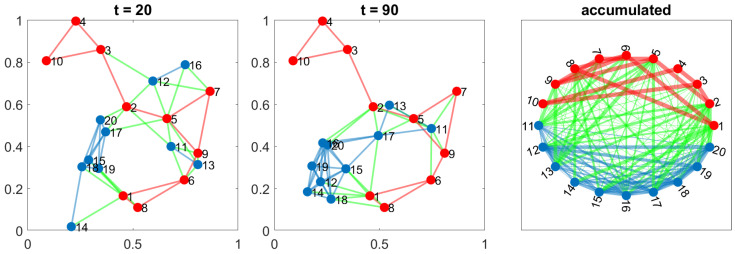
Network evolution with ten drones moving freely following cyclic random walks, combined with a set of 10 beacons in fixed positions. Red links represent the beacons’ network, the blue ones drones, and the green are auxiliary links between robots and beacons.

Any configuration can comprise one or more of these five scenarios. The movements of the agents on the orchard define a switching topology in the network, varying the set of neighbors for each agent at time $t,N_{i}\left(t\right)$ due to the entrance or leaving the detection range. Figure 3, Figure 4, Figure 5, Figure 6 and Figure 7 show the network at two instants (t=20 and t=70). Therefore, we can consider the entire graph G=∪G(t) a weighted graph, where G(t) is the network at time *t* and the weight $w_{ij}$ indicates how much time agents $a_{i}$ and $a_{j}$ have been connected. This is the third graph depicted in the figures mentioned above.

All experiments have been configured with the same parameters, except those related to the particular configuration of the experiment. Nevertheless, to clarify the experimental design and ease the comparison of the result, we include a summary of the parameters and any relevant assumptions made.

**Figure 8:**
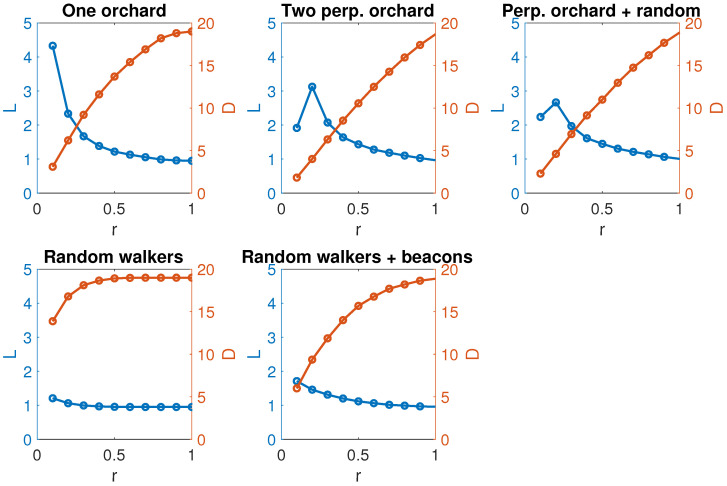
Evolution of the average degree and the shortest path length. (Figures corresponding to tests ordered from up to down and left to right).

- As the chosen topologies are RGGs, the area is defined as a unit square when just one orchard is simulated (tests 1, 4, and 5). For those tests involving two orchards (tests 2 and 3), the complete area is formed by two adjacent unit squares.
- Agents with rectilinear movement run with random speeds. Lanes are equispaced in their corresponding unit square.
- Random walks are generated with a variable speed in the interval (0,0.1) and a random angle in [0,2π]
- The static beacon network (test 5) is generated as an RGG with the same parameters as the dynamic networks and a connection radius r=0.3. (This radius provides the best performance, as can be seen in Figure 8).
- The weights in the aggregated network correspond to the number of times two agents have been connected.
- All tests run over 200 iterations.

#### 3.3.1. Test 1—One Orchard

It is defined by one set of agents moving in the same direction. Different speeds and an offset can be introduced. The connection radius between agents in adjacent lines must be large enough to reach at least one neighbor. Figure 3 shows the location of the agents at two different times and the corresponding GTG with a radius r=0.3. We accumulate all the connections and define the network as the union of all the networks at all analyzed epochs. The links’ weight indicates how often two agents have been inside their reception radius. The plot on the left depicts the final network structure, an open ring connecting the two closest neighbors.

#### 3.3.2. Test 2—Two Perpendicular Orchards

It follows a similar configuration as that in one orchard. It considers two adjacent fields, with lines of trees (and paths) distributed in perpendicular directions. As shown in Figure 4, the right-most agents in the vertical part act as bridges with all the agents with horizontal movements. The number of mutual connections will depend on the speed of agents, but eventually, all agents are susceptible to being connected. The aggregated final network can be considered as two open rings, where all the agents are connected with a subset of the right-most agents of the other ring depending on the radius r=0.3.

#### 3.3.3. Test 3—Two Perpendicular Orchards and a Free Agent

The last model extends test 2 with an agent that moves freely across the orchard. It can represent a drone or other kind of vehicle without constraints. This agent follows a random walk inside the boundaries of the complete orchard. This agent is marked in red in Figure 5. Depending on the agent’s movements and range, the resulting aggregated topology can vary, but some common properties emerge from this pattern, as seen in the following section.

Each tractor can be viewed as a periodic task, where the period is the time they take to go and return to the same place (start of their path). If we calculate the hyper-period as the least common multiple of the periods of such trips, one could study only that hyper-period to see whether all agents are connected or not.

#### 3.3.4. Test 4—All Drones Are Modeled as Random Walkers

There are cases where robots move freely through the orchard, like having a set of drones flying through the orchard. We use random walks to model their movement patterns. It is common for robots to return to their starting point to restart a new cycle, so a recurring pattern is used to take them back to their origin. Each agent will have its period, as in Test 3, so the study of the system up to the hyper-period guarantees the convergence of the consensus process.

The interest of this scenario is to test the performance of the algorithms in dynamic networks, to detect possible blind spots where synchronization is lost, and to compare it with other configurations where they are supported by other agents, as in Test 3 and Test 5.

In this scenario, agents move within a space of dimension 1 × 1 and connect to other agents within a radius of 0.3 (see Figure 6).

#### 3.3.5. Test 5—Drones with Static Beacons’ Network

One problem with cyclic random walks is that, due to the orchard configuration and movement patterns, there may be agents who become isolated or whose connection time with the rest of the network is not long enough to complete the consensus process.

This scenario examines the effect of a static beacon network that supports communication between mobile agents. The beacons are distributed throughout the space and are connected to form an RGG because it is one of the optimal, fault-tolerant structures that favor the performance of the consensus process.

Figure 7 shows the evolution of a possible configuration. The beacons’ network is represented in red. Blue links are the connections between the agents, and the green ones indicate a connection between an agent and one of the beacons. In the central plot, corresponding to instant t=90, we can see that agents 14 and 15 keep the connection with the rest of the network thanks to some of the available beacons around them.

#### 3.3.6. Comparing the Scenarios

Tests 1 to 5 describe the basic configuration of a swarm of vehicles moving across orchards. Actual scenarios can be formed by deforming the unit square areas (adapting the routes in consequence) and combining two or more of the cases. Therefore, we can consider any existing orchard as a mosaic of several elementary pieces according to their shape, orientation, and type of displacement (linear, free, and static).

To compare the five scenarios, we have analyzed the average degree (D) and the average shortest paths (L) of each type of aggregated network. They are relevant measures because the efficiency of the CoL process depends on them (not the resulting value, which is independent of the network topology). Networks with high degrees need to exchange information with more neighbors, which requires higher computational capabilities. On the other hand, longer paths provoke more extended periods until the consensus converges and a final training is obtained for the neural network.

The configuration for this experiment is the following one. Each case is composed of a total of 20 agents. The connection radius varies from 0.1 to 1 in steps of 0.1. Each configuration repeats 100 times and the degree and path lengths are averaged.

Figure 8 shows the variation of these magnitudes as the connection radius increases. The evolution for Tests 1 and 2 are similar: the path length decreases, and the degree increases with the radius. Nevertheless, in the third plot, we can see that the effect of the free agent is to keep both values almost constant. Its effect is to regularize the network degree and ease the connectivity of regions physically separated in the ground. The network generated from Test 4 (random walkers) remains almost constant concerning the connection radius, both for the average degree and the length of the paths. In the case of the paths, the network is almost complete from relatively small radii, but in return, the average degree is excessively high, which increases the cost of communications. In contrast, in Test 5, the network formed by the beacons makes it possible to maintain connectivity in relatively small networks while keeping the paths short.

Test 1 (one orchard) obtains the optimal configuration with a communication radius three times the distance between lanes. The network structure tends to be linear, and that topology increases the number of iterations needed for the consensus process to converge. Test 2 (two orchards) needs a higher communication range, probably due to the role of the nodes along the bounds between the two configurations. The obtained networks combine a densely connected group (even a clique) with a linear queue. Agents in the dense community usually agree faster, unless agents in the linear part differ significantly from the rest of the group. The free agents in Test 3 act as a hub, but barely affects the degree distribution and path length. It just reduces the required communication radius. Tests 4 and 5 present a completely different scenario. Probably it is due to the liberty of movement for the agents, which are not limited to a small area, but to the complete orchard. In both cases, we obtain quite dense topologies that imply a higher number of messages, leading to a possible communication overload. In the case of Test 5, the reduction in the required communication radius solves this problem partially and is conducive to better solutions.

**Figure 9:**
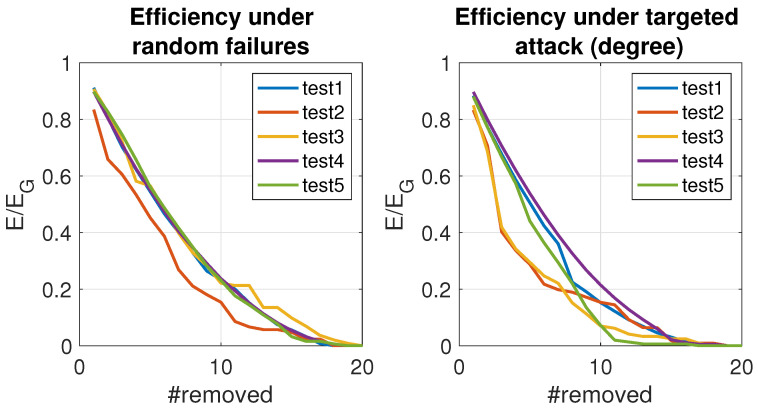
Efficiency of the scenarios under random failures and deliberate attacks. All networks created using a communication radius r=0.3.

Finally, the efficiency of the network has been studied to analyze the failure tolerance (see Figure 9). On the left, we can see no significant differences in the behavior under random failures. This is a consequence of being a GTG, where the spatial constraints avoid the existence of hubs, and the degree distribution follows a Poisson distribution instead of a power law one. Nevertheless, we can notice that the two-orchard scenario is slightly worse than the rest. This is due to the relevance of the agents along the boundaries. In this kind of configuration would be desirable to introduce some reinforcement.

However, the situation is different for deliberate attacks. When we block the best-connected agents, the efficiency of the network degrades quicker than in Test 1 and also in those involving random walkers. This is because the right-most agents of perpendicular orchards, or the free agent, play as a bridge, with a slightly higher degree than average. Beacons play a similar role in Test 5. That is why these agents are critical and precise in special surveillance. For targeted attacks, networks formed exclusively by agents following random walks are more robust than all the rest, as expected. In the case of Test 5, we can see how the performance of the network decreases clearly when most of the beacons are removed.

In general, we can assume that the intersection between D and L defines the most balanced radius connection. The inclusion of drones or beacons reduces the required distance among devices, offering a good enough performance for the consensus process. It allows a reduction in the communication radius, shortens the average path length, and has a good performance with a low degree, which translates into a reduction in the number of messages and a faster consensus convergence. Meanwhile, it maintains good efficiency under random failures and a reasonable one unless most of the beacons are unavailable due to a deliberate attack.

We can conclude that the inclusion of a network of static beacons and agents with free movement capacities improves significantly the performance of the consensus process to reach agreements in a swarm of autonomous vehicles.

## 4. Orchard Digital Model for Validating WANET Designs

The approach presented here supposes using a Digital Model of the orchard where the WANET is being deployed. At design time, it can be tested to ensure that the system deployed would be strongly connected.

A new version of the FIVE toolkit will be used to see in the virtual environment the dynamic evolution of the connections between the different agents as they are moving and entering or exiting the range of other agents’ wireless antennas.

The simulations generated will be executed during the hyper-period of the different agents, considering that the agents’ periods are the time they need to return to their initial position.

### 4.1. FIVE

The FIVE (Flexible Intelligent Virtual Environment designer) (https://github.com/FranEnguix/five, accessed on 1 February 2024) framework facilitates the creation and modification of a 3D IVE inhabited by SPADE (Smart Python Agent Development Environment) agents [7] effortlessly and expeditiously. This section presents a detailed overview of the FIVE framework and elaborates on the methodology used to model the environment. So, first, a description of the FIVE Designer system is presented, describing how an IVE can be easily created or modified. Next, the FIVE Execution System shows how the designed IVEs can be executed.

#### 4.1.1. FIVE Designer

**Figure 10:**
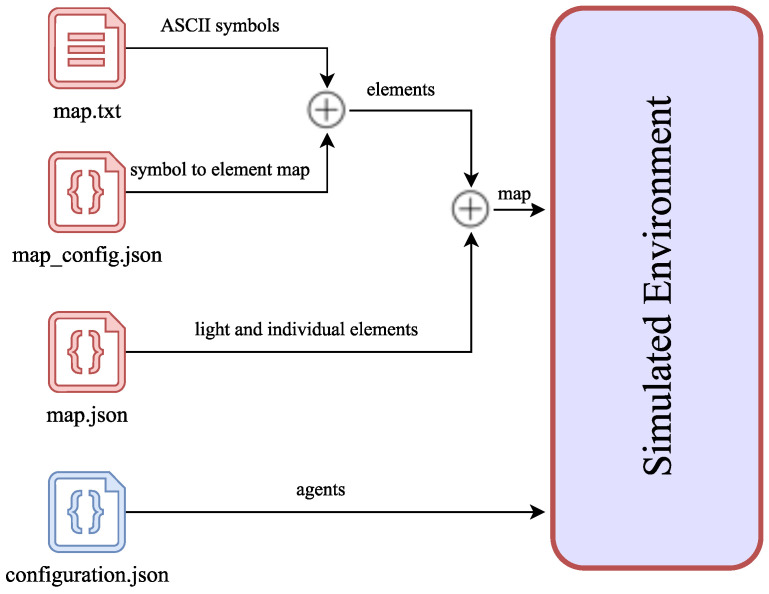
Files involved in the generation of the IVE and the agents.

The FIVE framework enables the creation of 3D environments using an integrated text-based map editor. Additionally, it offers the generation of custom agent avatars equipped with sensors, such as cameras. Defining a simulation is straightforward and efficient, requiring only modifying four text files to create the IVE and the agents (see Figure 10). Among these files, the first three red files (map.txt, map_config.json and map.json) are responsible for generating the IVE, which includes light objects, agent spawn points, and other relevant elements. On the other hand, the last blue file configuration.json) is utilized for generating the agents.

Once the constituent elements of the simulated IVE have been defined, the next step involves specifying the behavior of the inhabitant agents situated within the IVE. To achieve this, the FIVE framework incorporates a template for the inhabitant agents, composed of a SPADE agent with a Finite State Machine (FSM) behavior that governs the agent’s execution cycle.

After setting up the files and defining the behaviors of the agents to meet our requirements, the next step is to initiate the execution of the framework modules. The subsequent section will provide a comprehensive overview of the modules and outline the steps for successfully running the simulation.

#### 4.1.2. FIVE Execution System

The FIVE Execution System is a modular system that offers the flexibility to distribute computational load across multiple machines. It consists of three main components:

- The XMPP (Extensible Messaging and Presence Protocol) server enables communication between the agents and the environment.
- The FIVE Server Agent, which is developed using the Unity (Bellevue, WA, USA, https://unity.com, accessed on 1 February 2024) engine.
- A collection of SPADE inhabitant agents that populate the IVE.

To launch an application, the execution order must be as follows: first, execute the XMPP server (if the application is going to connect to a new one). Then, run the FIVE Server Agent. Furthermore, finally, execute the SPADE inhabitant agents.

The inhabitant agents and the Server Agent connect to the XMPP Server, as they will communicate through this protocol, and the FIVE Server agent interacts with the SPADE inhabitant agents via XMPP presence control and messages conveyed through a custom open-source protocol.

The inhabitant agents send commands to the environment (FIVE Server Agent), such as moving their avatar to a new position or capturing an image with their equipped camera. For this reason, the FIVE Server Agent should be the next module to be executed. These components can be run independently on different machines at different local networks.

**Figure 11:**
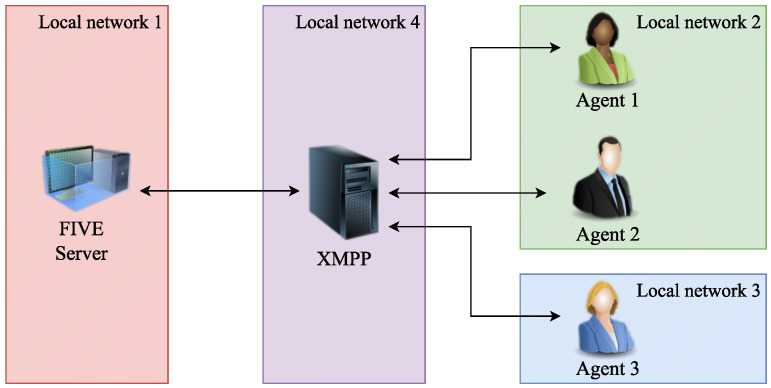
Example of FIVE architecture deployed.

Figure 11 illustrates a FIVE simulation deployed across four distinct local networks. The colored rectangles depict different local networks, and the arrows denote network sockets. Each agent portrayed in the figure operates on a different machine. Agent 1 and Agent 2 are situated on the same local network (Network 2), and all agents are connected to the XMPP server.

Based on SPADE, the agents exercise control over the virtual avatars that populate the IVE that the FIVE Server manages. The framework supports network failure tolerance, which allows for easy reconnection of agents to the FIVE simulator, thereby enabling the resumption of their activities.

The FIVE Server agent is in charge of the perception of inhabitant agents. As it has been extended for WANETs, the agents’ antenna ranges can be configured, and this range is used to perceive which other agents are in their range and send it to the inhabitant agent. Each inhabitant agent will be subscribed to the presence of all the agents in the multi-agent system. The agent will have a set of active neighbors ($N_{i}\left(t\right)$ in the CoL algorithm) that will change every time it receives the perception from the FIVE Server. Then, the agent will attend only to the presence of their current active neighbors.

## 5. Case Study: A Simulation of Fruit Orchard Smart Areas

In this case study, the agents control tractors moving through fruit orchards located next to each other. As the tractors move through the orchards, they can communicate with only the tractors inside their antenna range. As the tractors move, they can communicate with other agents depending on this range.

**Figure 12:**
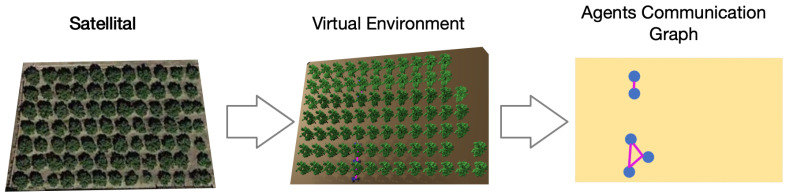
Several views of a fruit orchard.

Figure 12 shows three views of the same fruit orchard. On the left, there is a satellite capture. In the middle of the figure, we can see the representation of this orchard in FIVE. On the right is the schematic representation of the communications between the tractors.

**Figure 13:**
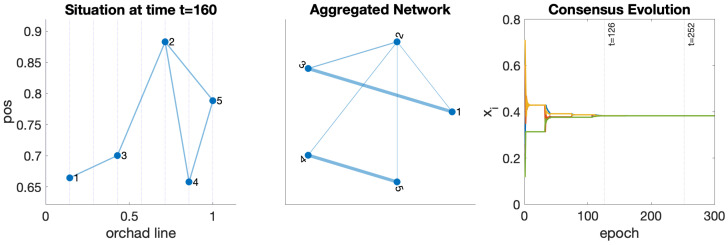
Result of the simulation allowing robot 2 to change between second and fifth lines in the orchard. (**Left**): situation at t=160. (**Center**): aggregated network. (**Right**): Evolution of the consensus over two hyper-periods.

Tractor 2 has double the speed of the rest. Two isolated groups appear if they follow the distribution shown in Figure 12. Allowing tractor 2 to change the orchard line, the team achieves the connection in one strongly connected component and the data propagation among all participants. Figure 13 shows the result of this scenario.

The simulation has a hyper-period t=126. The plot on the left depicts the situation of the yard in t=160. The one in the middle shows the aggregated network generated after the hyper-period. A stronger connection appears between stable teams {1,3} and {4,5}. Tractor 2 acts as a bridge between groups. Finally, the plot on the right is a sample of the consensus process over one common variable. The hyper-period and twice the hyper-period value are marked vertically. The mean absolute error at t=126 is $MAE=1.6\times 10^{-3}$, and when the second hyper-period completes at t=252, $MAE=8.8\times 10^{-6}$. So, this is when we can estimate that the team needs to complete one training epoch of the neural network, exchange the weights, and combine the models using CoL.

**Figure 14:**
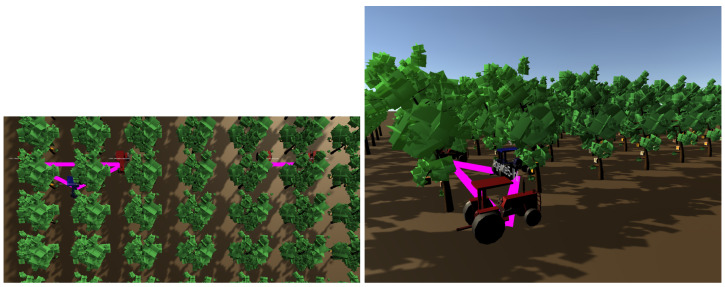
Overhead and side views of the fruit orchard.

Figure 14 presents two different views of the simulated fruit orchard in FIVE: the left image shows an overhead representation, whereas the right image shows a side view where an agent is represented as a tractor. These images show the same moment that is shown in the agent communication graph in Figure 12. So, in this last figure, the representation of the dynamic communication graph in the virtual environment can be observed as the edges (drawn in pink) appear and disappear, showing the availability of communication between agents in the antenna range of other agents. As observed in these figures, if all agents move at only one line, then two different components in the system will not communicate between them. Changing the system’s design, making that agent two moves along two other lines, would achieve the connection between both components.

## 6. Including Artifacts into FIVE

As can be observed in Test 5 in Section 3.3, it is essential in some cases to highlight the importance of some IoT devices that will be used as beacons to ensure the full connectivity of the network in this kind of systems. Due to this importance, it was decided to include artifacts to model such IoT devices when developing these systems. Artifacts, as discussed in Section 2.1, are passive, reactive parts of a MAS that model the environment with the services and functions the agents need. They can represent knowledge, information, or data in a structured and formal way, which can help to improve the efficiency and effectiveness of MAS.

These devices serve a specialized purpose as connection beacons and find applications in areas where the network signal quality is suboptimal. Designed to address such challenges, these devices establish a compact network infrastructure, providing a reliable means for agents to connect. In this network, agents can seamlessly exchange messages, fostering effective communication, and collaborate seamlessly to fulfill their assigned tasks. This enhances connectivity in environments with less-than-ideal signal conditions and facilitates the smooth coordination of activities among agents within the established network framework.

Although IoT devices are commonly connected through the MQTT protocol [51], we have opted for the use of the XMPP [52] protocol in our scenario. This communication protocol is open and extensible, serving as a platform for message exchange and network presence. XMPP is versatile, supporting instant messaging (IM), online presence, real-time collaboration, and various communication applications.

Operating on a client–server architecture, XMPP employs the XML (Extensible Markup Language) format for its message structure. It stands out as a federated protocol, enabling communication between XMPP servers and fostering interoperability across different services and networks. This characteristic has led to the development of a decentralized instant messaging network.

In contrast to the MQTT protocol, which prioritizes efficient message transmission in bandwidth-limited or unstable conditions, XMPP offers a different set of advantages. MQTT operates on a publish/subscribe messaging model, emphasizing efficiency and minimal resource usage. Notably lightweight, MQTT excels in environments where connectivity may be intermittent. In its publish/subscribe model, clients subscribe to topics and receive messages published in those topics.

Unlike XMPP, MQTT utilizes a more compact binary format, deviating from the XML format employed by XMPP. This divergence showcases the distinct strengths and focuses of each protocol, allowing users to choose the one that aligns best with their specific communication requirements and environmental constraints.

Incorporating artifacts within IVEs is pivotal in enriching the portrayal of projects integrating these components. These artifacts function as dynamic reservoirs leveraged by intelligent agents through interactive pathways. Notably, the FIVE system has undergone substantial advancements to streamline the creation of paired digital artifacts. Operating via XMPP, akin to SPADE agents, these digital entities can store and furnish pertinent data, encompassing metrics like temperature and humidity levels.

This evolution within the FIVE system facilitates the efficient utilization of digital artifacts and lays the groundwork for creating a Digital Shadow. This Digital Shadow serves as a comprehensive and evolving simulation of the virtual environment, integrating data from diverse real-world IoT artifacts. With the integration of IoT artifacts, FIVE transitions towards environments characterized by Digital Shadows, representing a more nuanced and dynamic reflection of the virtual world.

The artifact, bridging the physical realm, establishes a conduit with the agent engendered by FIVE, enabling the manipulation of correlated data within this virtual domain. Looking forward, FIVE’s trajectory extends beyond Digital Shadows, envisioning a future that progresses towards the realm of Digital Twins. This evolutionary path involves the creation of highly detailed and dynamic replicas of physical entities, mirroring their real-world counterparts with unprecedented accuracy.

Subsequently, the forthcoming section, notably within Section 6.1, will delve into the tangible application of this methodology within an Internet of Things (IoT) device. This specific device employs a regression model harnessed by a neural network (NN) to forecast temperature and humidity metrics. These prognostications are grounded in historical data encompassing temperature, humidity, and atmospheric pressure records.

The intertwining of the physical and digital realms, facilitated by digital shadow (DS) artifacts and the integration of machine learning models in IoT devices, engenders a more agile and anticipatory representation of physical phenomena within virtual landscapes. This convergence augurs pioneering prospects in the domains of modeling and simulation.

The incorporation of artifacts into FIVE is underpinned by the acknowledgment of the pivotal significance in forecasting and delineating the positioning of IoT devices during the design phase. This acknowledgment originates from the necessity to strategically ascertain the placement of key elements like antennas, which are instrumental in facilitating connectivity among scattered components. Integrating artifacts into FIVE directly responds to this pressing demand to accurately depict and manipulate IoT devices within their predetermined locations within the virtual realm.

The strategic necessity to integrate these components within FIVE was discerned throughout the case study. The capability to position and manipulate digital artifacts in precise locations, such as antennas for linking components, presents an invaluable advantage during the design phase. This empowers users to conduct more meticulous simulations and assessments of the interaction and efficacy of IoT devices within their proposed physical arrangement, thereby fostering well-informed and precise decision making in the initial stages of projects involving IoT devices.

### 6.1. Incorporating an IoT Artifact into the IVE

This case study represents a convergence of Internet of Things (IoT) devices, MAS artifacts, FIVE, and an NN for weather forecasting. The essential element of the project is an IoT artifact on an ESP32 M5Stack Core2. This versatile development kit boasts an array of features suitable for IoT applications, including WiFi connectivity for seamless data exchange with diverse online resources and services. This connectivity is crucial for our communication via the XMPP server with the FIVE system.

The IoT artifact is designed to forecast the temperature and humidity for the next hour based on a 24 h window of measurements encompassing temperature, humidity, and pressure. A Multilayer Perceptron (MLP) is trained using historical weather data to accomplish this. The network is then integrated into the ESP32 M5Stack Core2 artifact, facilitated by the tensorflowlite_esp32 library, enabling the deployment of TensorFlow Lite models on ESP32 microcontrollers.

Beyond the HTTP client functionality, the IoT artifact employs custom XMPP templates to communicate with the FIVE server. This communication mechanism allows the artifact to transmit its updated predictions to the server, contributing to a comprehensive and consistently updated weather model resource for the agents.

The project uses the FreeRTOS library, a real-time operating system for microcontrollers, to manage various tasks scheduled for execution over time. These tasks encompass sending updated predictions to the FIVE server, requesting normalized weather data from the Django endpoint, and consulting a Network Time Protocol (NTP) server to uphold accurate timekeeping.

### 6.2. Software Description

The dataset known as ‘Historical Hourly Weather Data’ (https://www.kaggle.com/datasets/selfishgene/historical-hourly-weather-data, accessed on 1 February 2024) constitutes an extensive compilation of weather information covering a period of approximately five years. This dataset offers hourly measurements of diverse weather attributes, including temperature, humidity, and air pressure. Beyond its utility for meteorological investigations, this dataset is a valuable resource for an array of interdisciplinary research endeavors.

We leverage this dataset to train the NN as a regression model. Obtained through the Weather API on the OpenWeatherMap website, this dataset is available under the ODbL License. It covers 30 cities in the US and Canada, along with 6 towns in Israel. Each weather attribute, such as temperature, humidity, and air pressure, is stored in a separate file, with rows representing the time axis and columns representing different cities. This organized structure facilitates ease of use and analysis. Additionally, for each town, the dataset provides information about the country, latitude, and longitude, which is a valuable asset for our project and empowers us to train the MLP-based regression model. This, in turn, enhances the capabilities of our IoT artifact.

**Figure 15:**
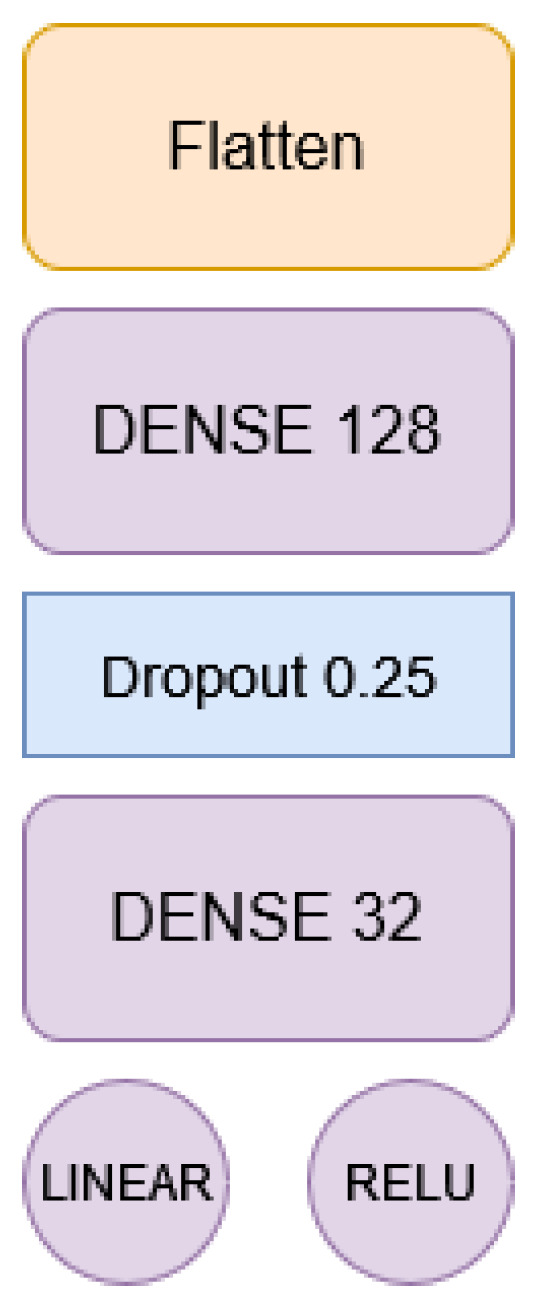
Top–down scheme of the Neural Network architecture.

We have developed a Keras MLP to construct the regression model. This model can use temperature, humidity, and pressure measurements from a 24 h window to predict the temperature and humidity for the subsequent hour. The NN’s architecture (Figure 15 comprises:

1. An initial flattened layer that reduces the input tensor (with dimensions 24×3) to a vector of 72 elements.
2. A dense layer of 128 neurons with a ReLU activation function, contributing 9344 parameters.
3. Dropout with a probability of 0.25 for better model generalization.
4. A dense layer of 32 neurons with a ReLU activation function, contributing 4128 parameters.
  - An output neuron with a linear activation function for temperature.
  - An output neuron with a ReLU activation function for humidity.

The network’s architecture involves 13538 parameters. Mean squared error (MSE) is the loss function on the validation set. The optimizer used is AdamW from the TensorFlow library, coupled with two callbacks: ReduceLROnPlateau, automatically adjusting the learning rate (LR) if there is no improvement over a specified period, and ModelCheckpoint, saving the best model obtained. Hyperparameters for ReduceLROnPlateau include an LR reduction factor of 0.1, patience of 30 units, and a minimum LR of $1\times 10^{-6}$.

The dataset has been refined to exclusively include temperature, humidity, and pressure data from Los Angeles, yielding 39,969 samples in the training set and 4442 samples in the test set, constituting a 90% train and 10% test split. Initial neural network hyperparameters include LR of $1\times 10^{-3}$, a batch size of 128, and 200 epochs. Extensive testing involving hyperparameter adjustments indicates that this configuration produces the most favorable results.

Subsequently, the NN is trained on Google Colab (https://colab.research.google.com, accessed on 1 February 2024), achieving an MSE of $7.5\times 10^{-4}$ on the test set. Following this, we employ the TFLiteConverter module to convert the decimal values of the neural network model weights into eight-bit integers, optimizing the model’s space and enabling seamless embedding within the IoT device.

### 6.3. Integration in FIVE

**Figure 16:**
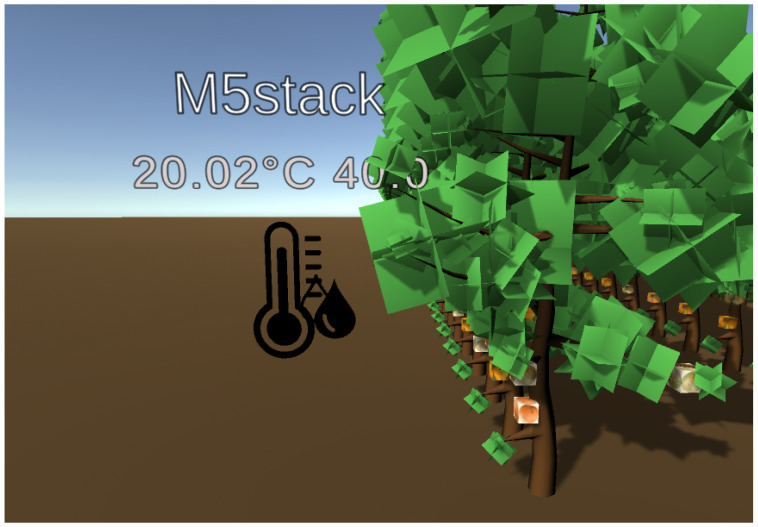
Aspect of the temperature and humidity IoT predictor device in the FIVE IVE.

The ESP32 M5Stack Core2 device is furnished with WiFi connectivity, enabling its utilization through the WiFi.h library. An XMPP template system has been devised to facilitate communication with the FIVE server. This system enables loading a foundational XMPP template containing the essential information required by the FIVE framework. It automatically supplements device-specific details, including the IoT device’s position within the IVE, the device type (in this instance, a temperature and humidity prediction device represented as an icon resembling a thermometer and two water droplets), and the device name displayed on its screen, as it is depicted in Figure 16.

Upon initiation, the IoT device initiates a connection to the WiFi network and the XMPP server. Subsequently, it configures the NTP server to synchronize with the current time. Following this, a command is dispatched to the FIVE server to create the artifact within the IVE. Utilizing FreeRTOS, the device executes a recurring task, named send_data_predict, at one-minute intervals.

The send_data_predict task comprises two phases. Initially, it retrieves the preceding 24 climate readings through an API developed on the Django server. Subsequently, the second phase involves converting the JSON data provided by the API into the float data type, followed by a conversion back to int8. These values serve as inputs for the developed NN, enabling predictions that are subsequently transmitted to the FIVE server.

## 7. Conclusions

This paper presented the application of *CoL* algorithm, a consensus-based decentralized Federated Learning algorithm, to dynamic Wireless Ad-hoc Networks and an extension of the FIVE framework with artifacts, for modeling IoT devices and validating the simulation.

This kind of system generates a Geographical Threshold Graph formed by artifacts and agents moving and being able to connect only with other entities that are inside their wireless antenna range. When designing these kinds of systems, it is essential to ensure that all agents are reachable at some time during the execution of the system. The paper proposes a method for testing this, generating a close-to-reality simulation of the environment (Digital Model) and executing the agents during the hyper-period of the duration of their routes (outward and return).

The paper proposes using the FIVE framework for easy creating and modifying the simulations and SPADE agents, as they are integrated into the framework. FIVE is used to design a Digital Model of a fruit orchard to test and validate the WANET design. It permits the visualization of the dynamic evolution of the network topology, where the ideas that have been commented on are exemplified.

In the paper, an evolution in how FIVE allowed us to model environments that approach the generated systems, enabling Digital Twins of the environment to be built, has also been presented. This is reached by including the concept of *artifact* in FIVE as a way of modeling the environment, allowing the development of a Digital Shadow of the environment modeled and a way to reach a Digital Twin quickly.

In future work, we also plan to explore different ways of optimizing the communication between the agents, such as using compression techniques, reducing the number of messages between the agents, or implementing selective model updates. Furthermore, we intend to address the question of how to optimize the use of energy and batteries in adaptive smart areas, as presented in [53,54]. Another future work direction is to evaluate the performance and effectiveness of CoL in different scenarios and applications where the agents may have different characteristics, objectives, and constraints, measuring the model’s accuracy, convergence rate, and communication overheads. Finally, we can explore integrating the Digital Twin concept with FIVE and testing CoL in a Digital Twin environment, enabling the agents to learn from real and virtual data and synchronize their models across the physical and digital domains. This could enhance the accuracy and robustness of the learning process, as well as the scalability and adaptability of the system. 

## Data Availability

No data available since experiments are randomized simulations.
